# Elevated S100A6 (calcyclin) enhances tumorigenesis and suppresses CXCL14-induced apoptosis in clear cell renal cell carcinoma

**DOI:** 10.18632/oncotarget.3169

**Published:** 2015-02-03

**Authors:** Xiang-Jun Lyu, Hong-Zhao Li, Xin Ma, Xin-Tao Li, Yu Gao, Dong Ni, Dong-Lai Shen, Liang-You Gu, Bao-Jun Wang, Yu Zhang, Xu Zhang

**Affiliations:** ^1^ Department of Urology/State Key Laboratory of Kidney Diseases, Chinese People's Liberation Army General Hospital/PLA Medical School, Beijing, People's Republic of China; ^2^ Department of Urology, Zhongnan Hospital, Wuhan University, Wuhan, People's Republic of China

**Keywords:** clear cell renal cell carcinoma, tumorigenesis, S100A6, CXCL14, apoptosis

## Abstract

Clear cell renal cell carcinoma (ccRCC) is often resistant to existing therapy. We found elevated S100A6 levels in ccRCC tissues, associated with higher grade pathological features and clinical stages in ccRCC patients. Knockdown of S100A6 inhibited cell proliferation *in vitro* and tumor growth *in vivo*. Gene expression profiling suggests a novel function of S100A6 in suppressing apoptosis, as well as a relationship between S100A6 and CXCL14, a pro-inflammatory chemokine. We suggest that the S100A6/CXCL14 signaling pathway is a potential therapeutic target in ccRCC.

## INTRODUCTION

Among all types of urological cancers, renal cancer is the second leading cause of cancer mortality in adults, and the clear cell renal cell carcinoma (ccRCC) is the most common histological subtype of renal cell cancer (RCC) [1]. Clear cell renal cell carcinoma is insensitive to chemotherapy and radiotherapy. Currently, the targeted therapies for the ccRCC are mainly focused on VEGF pathway and mTOR inhibition, however, the efficacy of targeted treatment is also limited [2, 3]. Therefore, the further insight into the molecular processes of ccRCC tumorigenesis and metastasis is needed to identify the promising prognosis or predictors and new potential therapy targets.

Calcium regulates a variety of intracellular processes through calcium-binding proteins. The S100 proteins family with two EF-hands has been observed in 69% (56 of 81) ccRCC and 70% (21 of 30) metastatic ccRCC [4]. Our previous microarray data (Gene Expression Omnibus code: GSE47352) about the gene expression in ccRCC tissues also demonstrated that S100A6 was highly elevated than other S100 protein members in the ccRCC tissues, including metastatic and non-metastatic. S100A6 is located in the chromosome 1q21, a region which frequently undergoes rearrangement, deletion, and translocation [5]. Several lines of evidence found that S100A6 level increased upon stress conditions such as ischemia [6], mechanical force [7], irradiation [8] or oxidative stress [9], which were most likely the tumor microenvironment. In fact, S100A6 has been found elevating in many types of cancers, such as melanoma [10], colorectal adenocarcinomas [11, 12], gastric cancer [13], pancreatic ductal adenocarcinoma [14, 15], astrocytoma [16], papillary thyroid carcinoma [17], choleteatoma [18], and osteosarcoma [19], and its important role in predicting the outcome and the possible therapeutic targets. However, the expression level and the molecular biology mechanism have not been discovered in clear cell renal cell carcinoma.

These observations and questions promoted us to investigate the role of S100A6 in ccRCC tumorigenesis and progression. In this study, we found with knock-down of S100A6, the tumor cells growth was suppressed *in vitro* and *in vivo*. In addition, we first reported a potential target gene of S100A6: CXCL14, a member of the CXC chemokine superfamily, involving in the inflammatory, autoimmune and tumorigenesis functions [20, 21], possessing chemoattractive activity for activated macrophages, immature dendritic cells, and natural killer cells [22]. Many independent studies have reported that CXCL14 was highly expressed in normal tissues, especially in normal kidney tissues, but absent in the tumor cell lines and primary tumors [23–25]. This characteristic is further proved by our study in the normal kidney and ccRCC cell lines. Many studies suggested CXCL14 as a tumor suppressor. In our study, we found that the inhibition of S100A6 can induce the expression of CXCL14 and cell apoptosis, and with knock-down CXCL14, the apoptosis of the cells are decreased. Together, these results suggest that elevated S100A6 enhances tumorigensis and suppresses CXCL14-induced cell apoptosis in clear cell renal cell carcinoma.

## RESULTS

### Expression of S100A6 is elevated in ccRCC tissues and correlates with pathological characteristics

**Figure 1:**
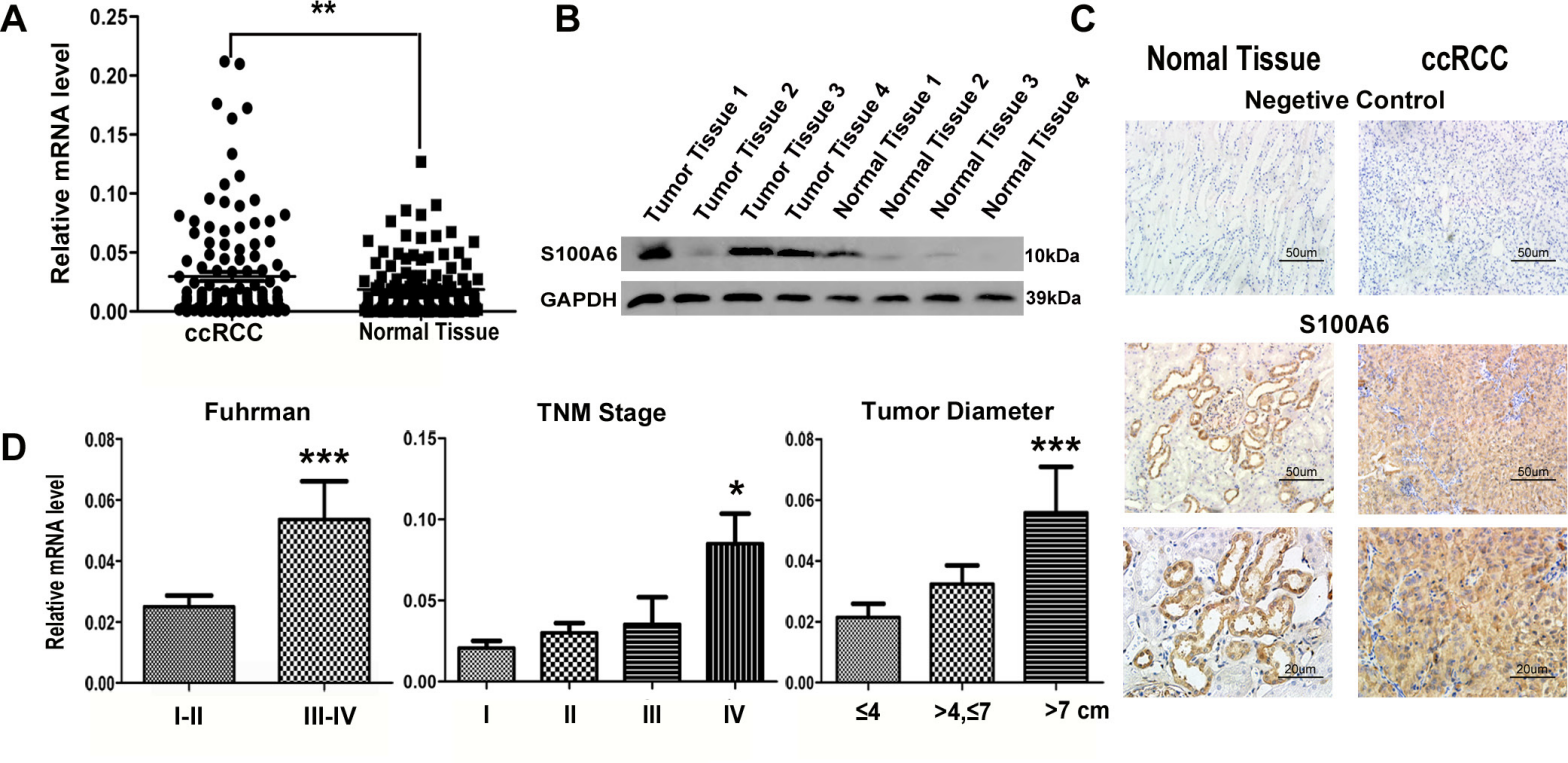
The elevated expression of S100A6 was detected in mRNA, protein and tissue of ccRCC samples The elevated S100A6 correlated with ccRCC pathologic and clinical characteristics. **(A)** Real-time PCR analysis of S100A6 mRNA levels in ccRCC (*n* = 129) and adjacent non-tumor tissues (*n* = 129). mRNA levels were normalized to PPIA expression. **(B)** Protein expression of S100A6 in paired samples of ccRCC and adjacent non-tumor tissues by Western blot analysis. GAPDH served as loading control. **(C)** Representative figures of the immunohistochemistry specimens of S100A6 in ccRCC and matched non-tumor tissues, and with negative controls in each group. **(D)** The total 129 ccRCC cases were divided into sub-groups according the Fuhrman Grade, TNM Stage and the maximum diameter of tumor. The Real-time PCR assay showed the relative S100A6 mRNA levels in each groups. All the Relative mRNA data were expressed as mean ± SD and comparisons were performed using Student's *t*-test. *p < 0.05; **p < 0.01; ***p < 0.001.

S100A6 has been reported to be highly up-regulated in many cancers [10–19], we thus examined the expression in ccRCC tissues and cell lines by quantitative RT-PCR (qRT-PCR) (Figure 1A), western blotting assay (Figure 1B), and immunohistochemical staining (Figure 1C). The tissue samples included 129 pathologically diagnosed ccRCC tissues paired with normal tissues adjacent to the tumor in each of the selected individual patients (Table 1). The clinicopathological features of the 129 patients and the tumor tissue samples detected were shown in Table 1. Our results showed that the mRNA levels of S100A6 were elevated in the tumor tissues than in the normal tissues (*P* < 0.01). Similarly, S100A6 protein levels were higher in tumor tissues compared to their normal counterparts (Figure 1B). In the immunohistochemical analysis, we found S100A6 was intensely expressed in both the cytoplasm and nucleus of ccRCC tissues than in the normal tissues.

**Table 1:** Clinicopathological features of the 129 patients and the tumor tissue samples

Variable	No. (%)	The mRNA expression of S100A6 (mean ± SD)	*P* value
**Gender**			***P* = 0.461**
Male	90(69.8)	0.028 ± 0.004	
Female	39(30.2)	0.033 ± 0.007	
**Age(year)**			***P* = 0.526**
≤40	22(17.1)	0.026 ± 0.009	
>40, ≤60	78(60.4)	0.033 ± 0.005	
>60	29(22.5)	0.023 ± 0.006	
**BMI**			***P* = 0.832**
<25	85(65.9)	0.025 ± 0.013	
≥25	44(34.1)	0.031 ± 0.022	
**Tumor size(cm)**			*P* = 0.0003***
≤4	62(48.1)	0.021 ± 0.004	
>4, ≤7	51(39.5)	0.030 ± 0.006	
>7	16(12.4)	0.054 ± 0.017	
**Fuhrman**			*P* = 0.037*
I	26(20.2)	0.021 ± 0.0047	
II	82(63.6)	0.026 ± 0.0045	
III	19(14.7)	0.048 ± 0.012	
IV	2(1.5)	0.109 ± 0.064	
**TNM Stage**			*P* = 0.0002***
I	106(82.1)	0.237 ± 0.004	
II	5(3.9)	0.029 ± 0.022	
III	10(7.8)	0.032 ± 0.015	
IV	8(6.2)	0.91 ± 0.016	
**Metastatic status**			*P* = 0.0014**
NM	111(86.0)	0.024 ± 0.004	
LM	8(6.2)	0.082 ± 0.024	
DM	10(7.8)	0.051 ± 0.016	

**TNM Stage**: TNM stage grouping was according to the 2009 TNM staging classification system. Stage I: T_1_N_0_M_0_, Stage II: T_2_N_0_M_0_, Stage III: T_3_N_0_(N_1_)M_0_, T_1_(T_2_)N_1_M_0_, Stage IV: T_4_N_0–2_M_0_, T_1–4_N_2_M_0_, T_1–4_N_0–2_M_1_. **Abbreviation**: BMI: body mass index; NM tumors involving non-metastasis; LM: tumors involving lymphatic metastasis; HM: tumors involving distant organ metastasis. Statistical analysis were described in Materials and Methods.

We compared the expressions of S100A6 among sub-groups of age, gender, BMI, Fuhrman grade, TNM stage, and tumor diameter (Figure 1D). No correlation existed between the expression of S100A6 and patients’ age, gender, or BMI. We divided the pathological groups and graded them into high-differentiation (Fuhrman grade I-II), moderate-differentiation, and low-differentiation (Fuhrman grade III-IV). S100A6 mRNA expression was lower in high-differentiation group than in the moderate- and low-differentiation (*P* < 0.001). When expression of S100A6 was compared among TNM stages, a stepwise upregulation of S100A6 was shown (Figure 1D). When tumors were further sub-divided based on the maximum diameter of tumors, we found that the expression of S100A6 was positively associated with the tumor diameter. Together, our data showed that the expression level of S100A6 had a significant correlation with tumor size, Fuhrman Grade, TNM stage and metastatic status (Table 1).

### Inhibition of S100A6 suppressed *in vitro* proliferation and *in vivo* tumor growth, and arrested cell cycle

**Figure 2:**
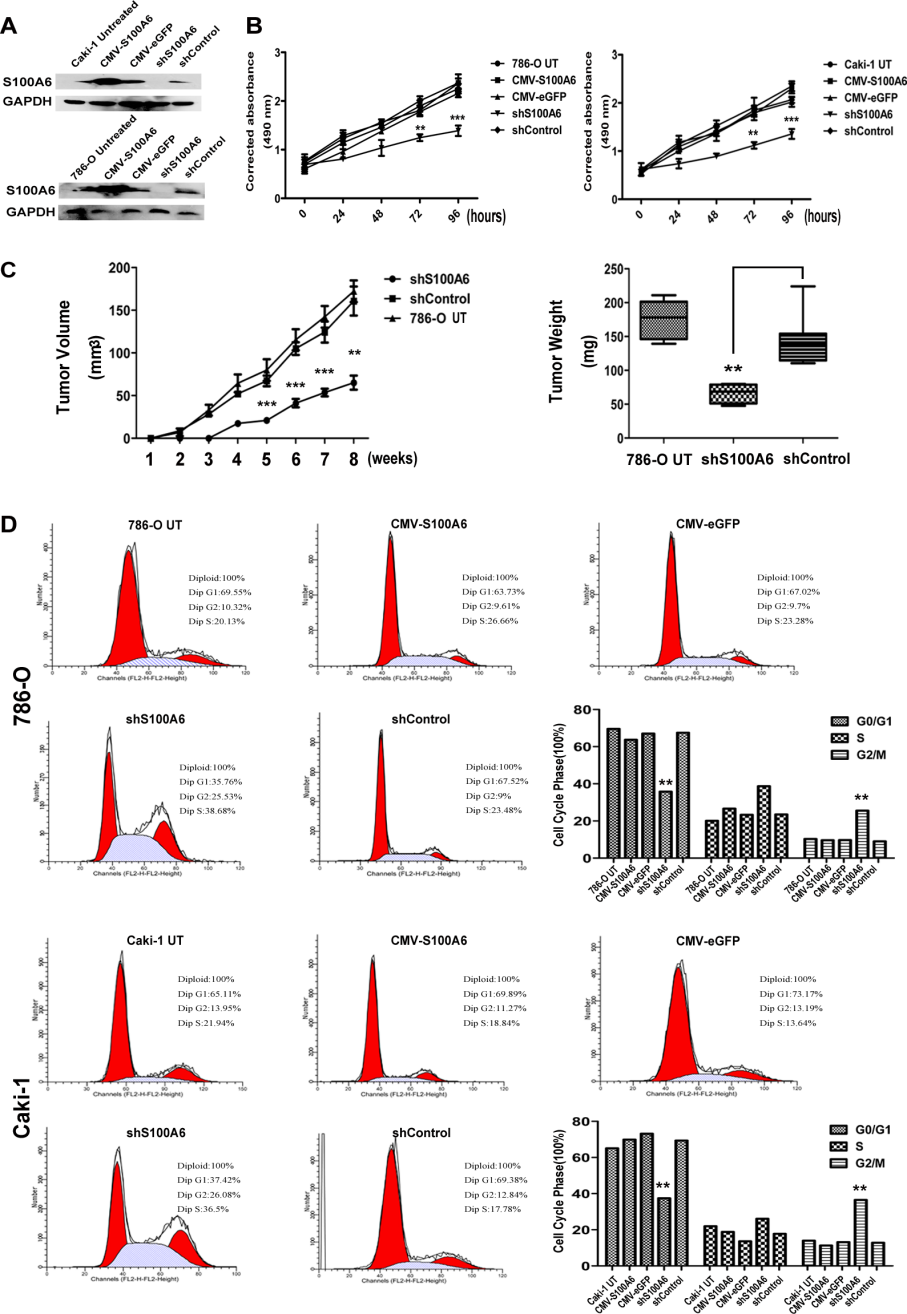
Inhibition of S100A6 suppressed cell proliferation *in vitro* and *vivo*, and effected the G2/M phase **(A)** Western blot assayed to identify the transfection efficiency of the 786-O and Caki-1 S100A6 stable cells, using the 786-O and Caki-1 untreated cells as control. The efficiency was satisfactory. **(B)** The proliferation curve by MTS assays showed that overexpression of S100A6 did not promote the 786-O and Caki-1 cells growth, whereas knockdown of S100A6 suppressed the cells growth. The data were expressed as mean ± SD. **(C)** Tumor growth curve of shS100A6, shControl and 786-O Untreated cells in nude mice. Tumor sizes were determined as described in the Materials and Methods. The tumor weight derived from the shS100A6 group was lower than from the shControl and 786-O untreated groups when nude mice were sacrificed at 8 weeks after injection. The data show as the min to max of tumor weight. **(D)** Influence of S100A6 in 786-O and Caki-1 cells on cell cycle distribution. 786-O and Caki-1 cells were stable transfected by overexpression and knockdown of S100A6, compared to vector control respectively and untreated cells control. The overexpression of S100A6 in both cell lines showed no differences in cycling phase distribution comparing to the empty vector control. The shS100A6 groups in both cell lines showed a lower percentage in G0/G1 phase and a higher percentage in G2/M phase comparing to the shControl and untreated cells control. *p < 0.05; **p < 0.01; ***p < 0.001.

To explore the biological mechanism of S100A6 elevated in ccRCC, we knocked down and overexpressed S100A6 in two ccRCC cell lines, 786-O and Caki-1. The efficiency of stable transfection S100A6 was detected in both cell lines by Western blotting analysis (Figure 2A). The vector maps were shown in Supplementary Figure 1A and 1B. The efficiency of transfection was shown in Supplementary Figure 1C and 1D. The cell growth of shS100A6, shControl, CMV-S100A6, CMV-eGFP and two untreated cell lines were measured by MTS assay at the time points of 0 h, 24 h, 48 h, 72 h, and 96 h. The results revealed that knockdown of S100A6 suppressed cell growth, while overexpression of S100A6 did not promote the 786-O, and Caki-1 cell growth (Figure 2B).

To determine whether knockdown of S100A6 suppressed tumorigenesis *in vivo*, 786-O cells stably expressing shS100A6, shControl, and untreated were suspended in PBS with an equal volume of Matrigel (BD Biosciences, USA) and injected in the left armpit of 5–6 weeks old BALB/c nude mice. Tumor volumes were measured with calipers every week after injection. The palpable difference of tumor growth was observed from two weeks after inoculation. The growth of tumors in animals injected with shS100A6 cells was distinctly slower compared with the animals injected with shControl, and untreated cells in the fifth weeks (Figure 2C). In the group of animals injected with shS100A6, only 4 out of 8 mice developed tumor, and the rest developed visible tumors 1 week later than mice in the 2 control groups. At 8 weeks after injection, the mice were sacrificed and the tumors were removed. The weight of tumors was smaller in the shS100A6 group compared with the control groups. The mice model was shown in Supplementary Figure 2A and HE straining of tumor was shown in Supplementary Figure 2B. Thus, we found that the knockdown of S100A6 suppressed cell and tumor growth *in vivo*.

Since S100A6 is a calcium-binding protein involving in the regulation of cell cycle progression, in ccRCC cell lines, knockdown of S100A6 inhibited cell cycle at the G2/M phase, in the 786-O and Caki-1 cell lines compared with the empty vector control and untreated cells. However, overexpression of S100A6 did not effect the cell cycle (Figure 2D).

### Microarray and bioinformatics analysis revealed the correlation between S100A6 and chemokine pathway, and directly regulation of CXCL14

**Figure 3:**
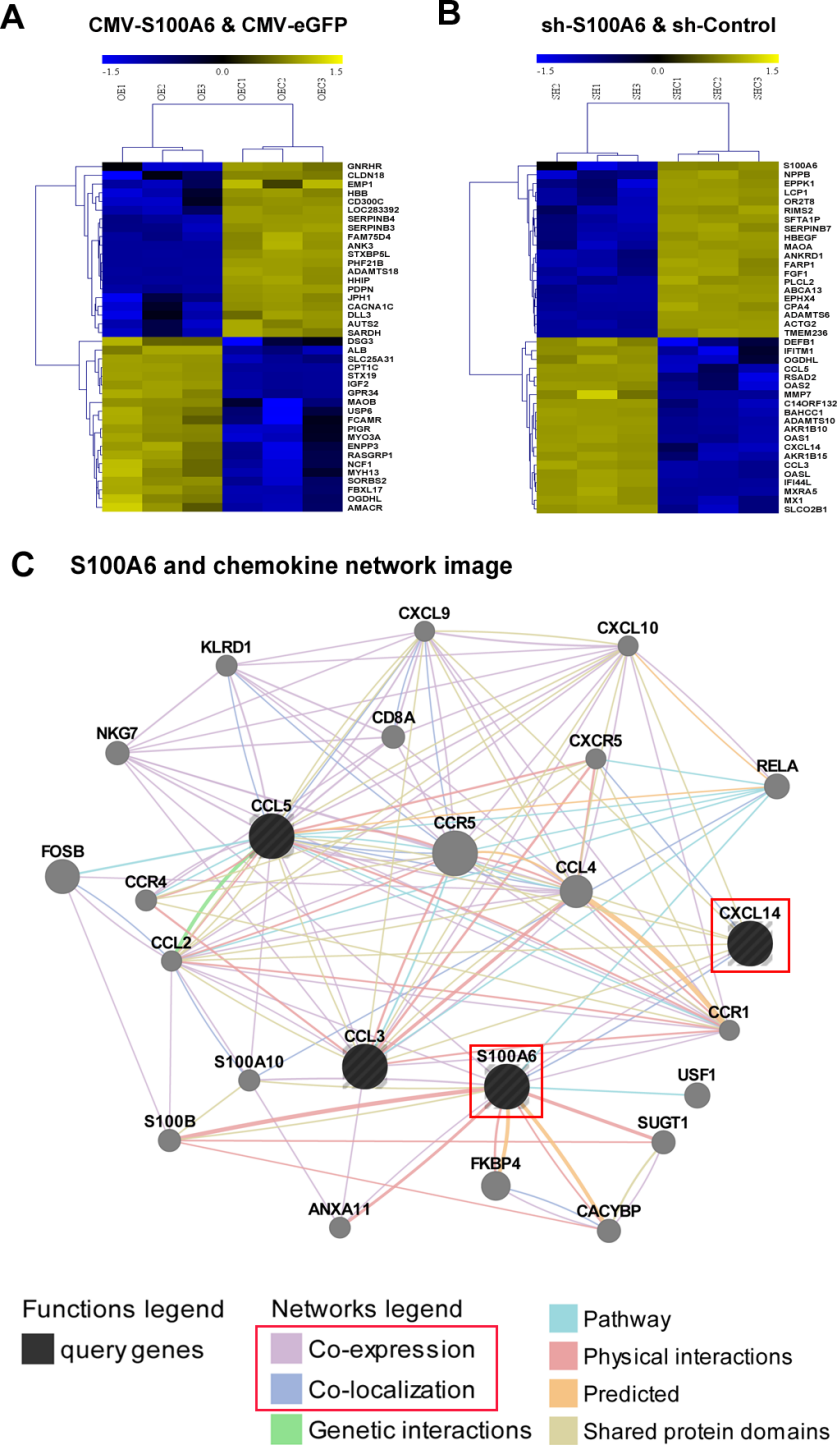
Microarray and bioinformatics analysis revealed S100A6 forceful associated with chemokine pathway, and regulated CXCL14 Microarray of mRNA expression profiling of the TOP 20 up-regulated and down-regulated different expression genes in group CMV-S100A6 and CMV-eGFP **(A)** and in group shS100A6 and shControl **(B)** in clear cell renal cell cancer cell line 786-O. **(C)** The network image of S100A6 and chemokine was analysis by GeneMANIA, and found the direct relation between S100A6 and CXCL14: Co-expression and Co-localization.

To understand the potential role of S100A6 in ccRCC, we performed cDNA microarray profiling in 786-O cells with either S100A6 overexpression or knockdown. Each biochip had tri-biological repeat to confirm the results. The top 20 up-regulated and down-regulated mRNA of two groups were listed in Figure 3A and 3B. A total of 344 differentially expressed genes (DEGs) were detected and shown statistical significance in the overexpression groups. As S100A6 was testified as an oncogene of ccRCC in our study, we focused on the knockdown groups. Approximately1399 DEGs were found between the 2 groups, of which 880 DEGs were up-regulated and the other 519 were down-regulated. In GO enrichment analysis, the top 12 clusters had tumorigenicity- and malignancy-related functions such as cell adhesion, cell differentiation, regulation of cell proliferation, and cytokine-mediated signaling pathway in GO Biological Process analysis and oxidoreductase activity, and growth factor activity in GO Molecular Function (Supplementary Table S3). The pathways analysis confirmed the results that the top 10 clusters focused on the cancer-related pathways (Supplementary Table S4). The statistics of top 20 DEG fold changes including the up-regulated and down-regulated DEGs in the knockdown groups are shown in Tables 2 and 3. We found that the top 20 fold changes DEGs were more involved in the chemokine signaling pathway, specifically the up-regulated DEGs (CCL5, CXCL14, and CCL3). The interaction and network among these 3 genes and S100A6 were further searched and predicted in GeneMANIA and there was direct correlation of co-expression and co-localization between S100A6 and CXCL14 (Figure 3C).

**Table 2:** List of 20 up-regulated mRNA after stable transfection of shRNA against S100A6

Fold change	*P*	Gene bank ID	Gene name (symbol)	Putative cellular function
40.556	0.0000	NM_005218	defensin, beta 1 (DEFB1)	immune response
39.076	0.0000	NM_003641	Interferon induced transmembrane protein 1 (9–27) (IFITM1)	protein binding
24.768	0.0003	NM_018245	oxoglutarate dehydrogenase-like (OGDHL)	protein binding
22.529	0.0002	NM_002985	chemokine (C-C motif) ligand 5 (CCL5)	chemokine activity
19.923	0.0000	NM_080657	radical S-adenosyl methionine domain containing 2 (RSAD2)	catalytic activity
17.773	0.0000	NM_016817	2′-5′-oligoadenylate synthetase 2, 69/71kDa (OAS2)	ATP binding
14.228	0.0000	NM_002423	matrix metallopeptidase 7 (matrilysin, uterine) (MMP7)	zinc ion binding
13.427	0.0025	NR_023938	chromosome 14 open reading frame 132	protein binding
13.378	0.0014	NM_001080519	BAH domain and coiled-coil containing 1 (BAHCC1)	DNA binding
12.735	0.0000	NM_030957	ADAM metallopeptidase with thrombospondin type 1 motif, 10 (ADAMTS10)	molecular_function
11.782	0.0000	NM_020299	aldo-keto reductase family 1, member B10 (aldose reductase) (AKR1B10)	protein binding
11.751	0.0000	NM_002534	2′-5′-oligoadenylate synthetase 1, 40/46kDa (OAS1)	ATP binding
11.739	0.0002	NM_004887	chemokine (C-X-C motif) ligand 14 (CXCL14)	chemokine activity
11.151	0.0000	NM_001080538	aldo-keto reductase family 1, member B15 (AKR1B15)	oxidoreductaseactivity
11.085	0.0017	NM_002983	chemokine (C-C motif) ligand 3 (CCL3)	chemokine activity
10.806	0.0001	NM_003733	2′-5′-oligoadenylate synthetase-like (OASL)	ATP binding
10.283	0.0000	NM_006820	interferon-induced protein 44-like (IFI44L)	immune response
10.214	0.0002	NM_015419	matrix-remodelling associated 5 (MXRA5)	not described
10.014	0.0022	NM_002462	myxovirus (influenza virus) resistance 1, interferon-inducible protein p78(MX1)	GTP binding
9.961	0.0001	NM_007256	solute carrier organic anion transporter family, member 2B1 (SLCO2B1)	transporter activity

**Table 3:** List of 20 down-regulated mRNA after stable transfection of shRNA against S100A6

Fold change	*P*	Gene bank ID	Gene name (symbol)	Putative cellular function
116.959	0.0000	NM_014624	S100 calcium binding protein A6 (S100A6)	calcium ion binding
54.815	0.0000	NM_002521	natriuretic peptide B (NPPB)	hormone activity
14.612	0.0000	NM_031308	epiplakin 1 (EPPK1)	structural molecule activity
11.048	0.0000	NM_002298	lymphocyte cytosolic protein 1 (L-plastin) (LCP1)	actin binding
10.011	0.0001	NM_001005522	olfactory receptor, family 2, subfamily T, member 8 (OR2T8)	olfactory receptor activity
9.908	0.0002	NM_014677	regulating synaptic membrane exocytosis 2 (RIMS2)	Rab GTPase binding
9.662	0.0002	NR_027082	surfactant associated 1, pseudogene (SFTA1P)	not described
8.966	0.0000	NM_001040147	serpin peptidase inhibitor, cladeB (ovalbumin), member 7 (SERPINB7)	regulation of proteolysis
8.941	0.0000	NM_001945	heparin-binding EGF-like growth factor (HBEGF)	growth factor activity
8.273	0.0000	NM_000240	monoamine oxidase A (MAOA)	primary amineoxidaseactivity
7.807	0.0000	NM_014391	ankyrin repeat domain 1 (cardiac muscle) (ANKRD1)	p53 binding
7.309	0.0000	NM_001145014	ret finger protein-like 4A (FARP1)	cytoskeletal protein binding
7.243	0.0000	NM_000800	fibroblast growth factor 1 (acidic) (FGF1)	S100 protein binding
6.578	0.0000	NM_015184	phospholipase C-like 2 (PLCL2)	calcium ion binding
6.555	0.0001	NM_152701	ATP-binding cassette, sub-family A (ABC1), member 13 (ABCA13)	ATP binding
6.425	0.0000	NM_173567	epoxide hydrolase 4 (EPHX4)	hydrolase activity
6.345	0.0000	NM_016352	carboxypeptidase A4 (CPA4)	zinc ion binding
6.276	0.0000	NM_197941	ADAMmetallopeptidasewiththrombospondintype1motif,6(ADAMTS6)	metallopeptidase activity
6.189	0.0000	NM_001615	actin, gamma 2, smooth muscle, enteric (ACTG2), transcript variant 1	ATP binding
5.877	0.0349	NM_001098844	transmembrane protein 236 (TMEM236)	not described

### S100A6 knockdown up-regulates CXCL14

**Figure 4:**
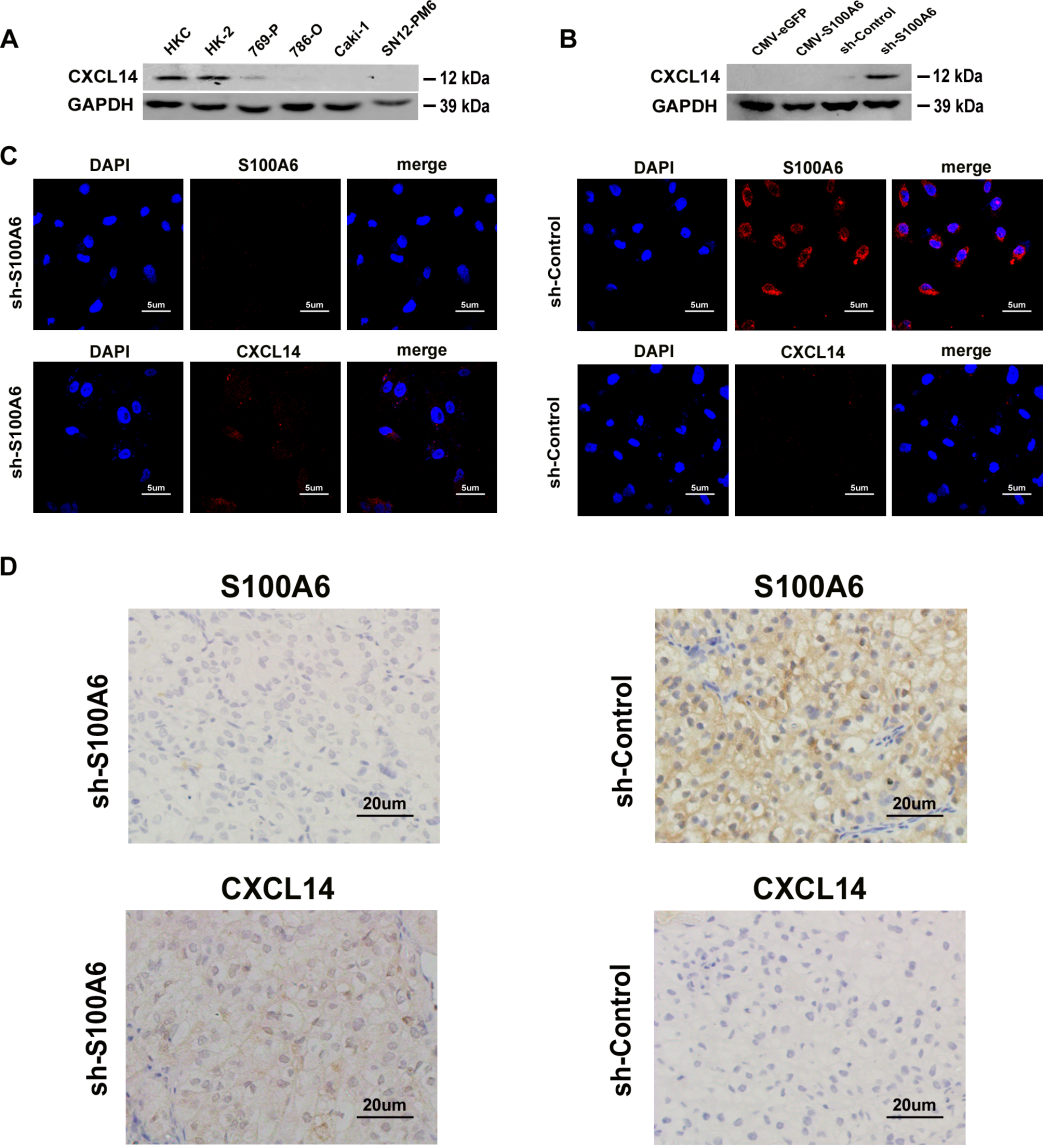
S100A6 was co-expression and co-localization with CXCL14 in cells and the subcutaneous xenograft tumor tissues **(A)** The protein expression of CXCL14 in cell in two normal kidney cell lines: HKC and HK-2, as well as four ccRCC cell lines: 769-P, 786-O, Caki-1 and SN12-PM6 were detected by Western blot analysis. **(B)** The protein expression of CXCL14 of different transfected S100A6: the overexpression and knockdown of S100A6, together with each empty vector of 786-O were also detected by Western blot analysis. **(C)** The expression and location of S100A6 and CXCL14 were analyzed by immunofluorescence staining in 786-O cell line. **(D)** The expression and location of S100A6 and CXCL14 were analyzed by immunohistochemistry in the subcutaneous xenograft tumor tissues of shS100A6- and shControl-injection groups. CXCL14 was strongly stained in the tissues which were developed from the shS100A6 cells.

As the microarray and bioinformatics analysis showed knockdown of S100A6 promoted the expression of chemokine, and S100A6 demonstrated direct relation with CXCL14, we detected the expression of CXCL14 in 2 normal kidney cell lines and 4 ccRCC cell lines and found that CXCL14 was widely expressed in normal kidney cells, while barely expressed in tumor cells (Figure 4A). However, when S100A6 knockdown occurred in 786-O cells by stable transfection of shRNA, CXCL14 expression was restored, but was absent in cells overexpressing S100A6 (Figure 4B). Similar results were obtained by immunofluorescence analysis. As shown in Figure 4C, the staining of CXCL14 in the shS100A6 of 786-O cells were stronger in shS100A6 compared with the shControl cells. Both S100A6 and CXCL14 are located in cytoplasm and nucleus (Figure 4C).

To further confirm the correlation, we detected the expression of CXCL14 and S100A6 in the subcutaneous xenograft tumor tissues by immunohistochemical analysis (Figure 4D). In the tumor of shS100A6-injection group, the expression of CXCL14 in the tumor tissue was obviously stronger than in the tumor tissue of shControl-injection group. The above results confirmed the co-expression and co-location correlation between S100A6 and CXCL14.

### Knockdown of S100A6 activated CXCL14-induced apoptosis

**Figure 5:**
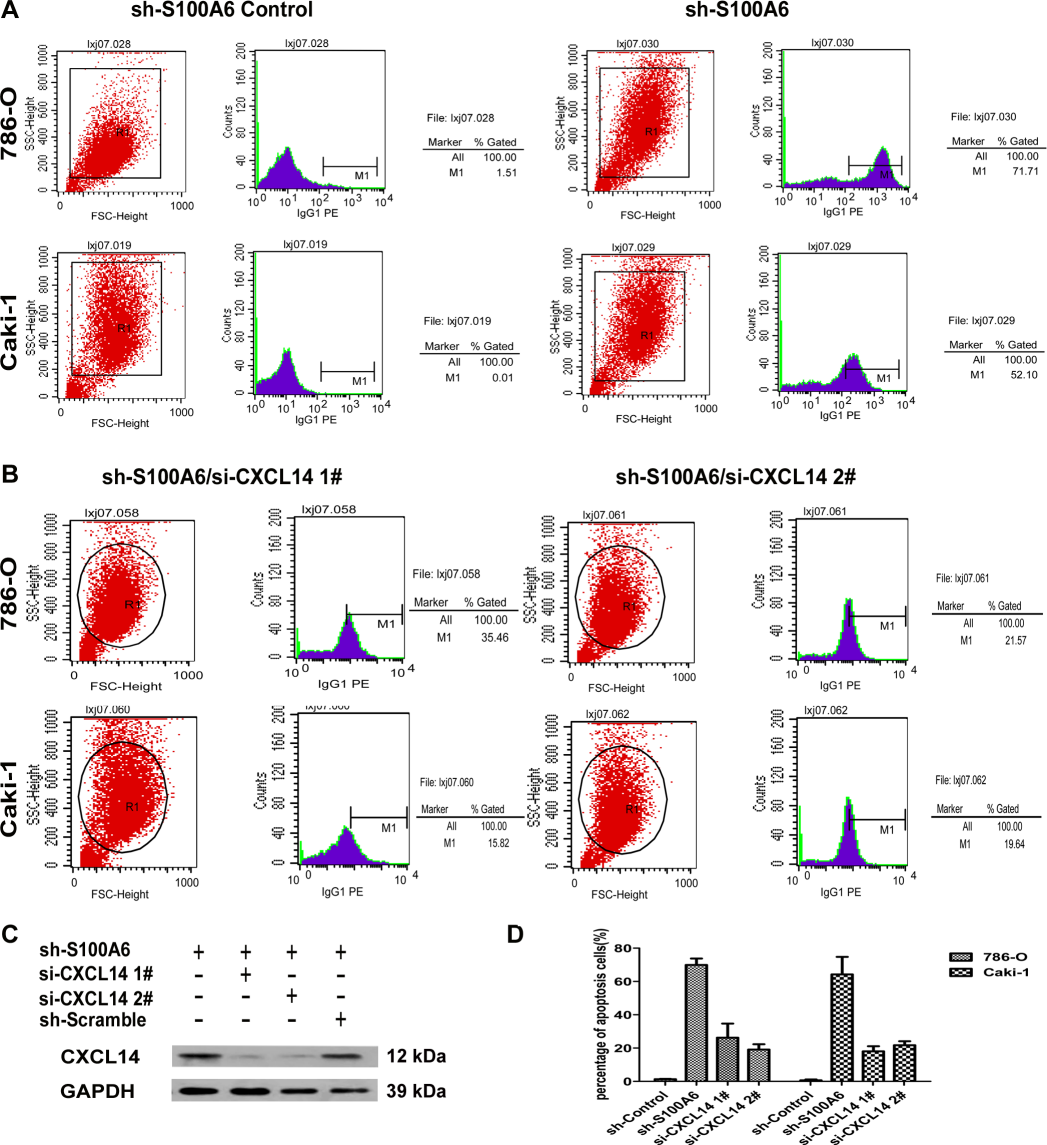
Knockdown of S100A6 activated CXCL14-induced apoptosis **(A)** The two-channel cell apoptosis analysis of shControl and shS100A6 in 786-O and Caki-1 cells detected the apoptosis in the screen eGFP live cells. **(B)** Further apoptosis analysis was performed in the shS100A6 cells which also knockdown the CXCL14, and the percentage of apoptosis were decreased. 1# and 2# were using the different CXCL14 si-sequence. **(C)** The transfection efficiency of si-CXCL14 1# and 2# in shS100A6 786-O cells were analysis by Western blot. **(D)** The percentage of apoptosis cells of shS100A6/si-CXCL14 1# and 2# were lower comparing to the untreated shS100A6 in both 786-O and Caki-1 cells. Data were expressed as mean ± SD and comparisons were performed using Student's *t*-test. *p < 0.05; **p < 0.01; ***p < 0.05

Because of the correlation and regulation of chemokine, we examined whether S100A6 promoted tumor growth by inhibition of ccRCC cell apoptosis. We created the transfection of si-CXCL14 cells using 2 si-sequence, 1# and 2# and analyzed apoptosis in the different treated cells by 2-channel flow cytometry analysis which only detected cell apoptosis in the marked screen eGFP live cells. The analysis of shControl and shS100A6 in 786-O and Caki-1 cells showed that the percentage of apoptotic cells dramatically increased in the shS100A6 for both cell lines from 1.51% to 71.71% and 0.01% to 52.1%, respectively (Figure 5A). To identify whether attenuation of S100A6 activated the CXCL14-induced apoptosis, we knocked down the CXCL14 with two siRNAs. The efficiency of transfection was detected 72 h after si-CXCL14 in the shS100A6 by the Western blot (Figure 5C) in 786-O cell lines. When knockdown of CXCL14 in shS100A6 in both 786-O and Caki-1 cells, the percentage of apoptotic cells was found to be decreased to 21.57% and 15.82%, respectively. Both 1# and 2# si-sequence showed the similar results (Figure 5B). Taken together, the percentage of apoptosis cells in the shS100A6 cells were dramatically increased compared to the shControl cells, and when knock-down of CXCL14 in shS100A6 cells, the percentage of apoptosis cells were decreased (Figure 5D).

## DISCUSSION

To our knowledge, this is the first report that reveals the expression of S100A6 and CXCL14 in clear cell renal cell carcinoma and indicates a role of S100A6 in ccRCC correlating with CXCL14. In this study, we explored that S100A6 was highly expressed in ccRCC, enhanced tumorigenesis of ccRCC and was very likely to suppress CXCL14-induced apoptosis. In the molecular and biological behavior assays *in vitro* and *in vivo*, the shS100A6 cells proliferation was suppressed. The stepwise up-regulation of S100A6 in the pathological feature such as diameter may due to the function of tumorigenesis enhancement and apoptosis suppression. S100A6 silencing induced G2/M phase arrest to suppress cell proliferation, which was also supported by previous studies showing that S100A6 depletion in endothelial cells increased the proportion of G2/M phase of the cell cycle [26]. However, in the NIH 3T3 fibroblasts cells, the depletion of S100A6 blocked the G0/G1 phase [27], Thus, S100A6 may have tissue-specific function in different type of cells. In this study, overexpression of S100A6 did not promote the proliferation. That may because the expression of S100A6 in the ccRCC cells was saturated for carrying on its function of enhancing tumorigenesis, the effect of the molecular and biological behavior was not evident. Hence, we concentrated our focus more towards the DEGs in the treatment groups transfected with shS100A6 and shControl. After microarray and bioinformatics analysis of the post-transfection of S100A6 shRNA, in the top 20 up-regulation DEGs, there were 3 chemokines: CCL5, CXCL14, and CCL3 showing more than 10 fold up-regulation. As the interaction of S100A6 and chemokine has not been reported previously, we built a gene network through the GeneMANIA search, and found that there was a potential and direct interaction between S100A6 and CXCL14. CXCL14 chemo-attracts iDCs (immature dendritic cells), and causes the functional maturation of dendritic cells, which is critical for tumor immunity, by up-regulating the expression of dendritic cell maturation markers. This in turn causes the proliferation of allogeneic T cells, which substantially contributes to the anti-tumor immune surveillance [28]. The functions of CXCL14 such as to attract the natural killer cells, activate monocytes to the sites of inflammation or malignancy, enhance recruitment of surrounding immune cells, and induce apoptosis [29, 30]. Previous studies have demonstrated that CXCL14 was epigenetically silenced in some cancers such as lung adenocarcinoma and prostate cancer. Results demonstrated that CXCL14 restoring led to a decreased in tumorigenesis [31, 32]. These previous findings were consistent with our results and provided important evidences of the roles which CXCL14 played in the cancer progression.

Although we proved the co-expression and co-location relation between S100A6 and CXCL14, the exact molecular biologic mechanism still has not been established. We searched the public data and bioinformatics website, and proposed a hypothesis that S100A6 may regulate CXCL14 through estrogen receptor 1 (ESR1). In Hela cells, S100A6 was reported to bind the nuclear ESR1 [33]. Another study found that the nuclear ESR1 can transcriptionally regulate the CXCL14, and activate its expression [34]. Thus, we conjectured that excess S100A6 competitive combination with ESR1, causing the decrease of free ESR1 expression, or somehow inducing the changes of molecular structure and genotype of ESR1. Therefore, CXCL14 cannot be transcriptionally regulated and the expression of CXCL14 was decreased, even absent. This possible inference will be our next research focus.

In this study, we have demonstrated that highly elevated S100A6 regulated clear cell renal cell carcinoma growth and progression. S100A6 promoted ccRCC tumourigenesis through influencing the cell cycle phase and suppressing CXCL14-induced apoptosis. We found and testified the correlation between S100A6 and CXCL14, and propose a hypothesis about the link point of ESR1. However, extensive experiments are needed to verify the precise pathway. Moreover, the mechanism about why the S100A6 elevated in ccRCC tissues is also unknown and needs further studies. In conclusion, the results of our study might be beneficial in the tumourigenesis mechanism of ccRCC, and might provide a valuable focal point in the development of ccRCC therapeutic strategies.

## MATERIALS AND METHODS

### Human tissues specimens

This study was approved by the ethics committee of the Chinese People's Liberation Army (PLA) General Hospital. A total of 258 ccRCC tissue specimens were obtained from 129 patients who underwent partial or radical nephrectomy at the Chinese PLA General Hospital from 2008 to 2012 and the pathological diagnosis was confirmed by a senior pathologist. Written informed consent was obtained from all the patients included in the study before the surgery. The TNM (Tumor Node Metastasis) stages of the specimens were assigned according to the 2009 TNM staging classification system [35]. The histopathological classification of tumor grades was performed according to Fuhrman. All the specimens were immediately snap-frozen and retained fresh after surgical removal. The patient demographics are given in Table 1.

### Cell lines

The normal renal cell lines: HKC, HK2, and the RCC cell lines: 786-O, 769-P, and Caki-1 were purchased from the National Platform of Experimental Cell Resources for Sci-Tech (Beijing, China) in the year 2012. The SN12-PM6 cell line was kindly provided by Dr. XP Zhang of the Department of Urology, Union Hospital, Wuhan, China. Detailed information of the cell cultures and reagents are listed in the Supplementary Data.

### RNA isolation and real-time RT-PCR

These methods were described previously [36] and detailed elucidated in Supplementary Data. The primers used in the PCR analysis were presented in Table S1.

### Protein extraction and western-blot analysis

Protein extraction and Western-blot analysis were performed using standard techniques described previously [37] and in Supplementary Data.

### Immunohistochemistry

The method was described previously [38] and in Supplementary Data.

### Plasmid constructs and transfection

The plasmids were constructed, and the stable transfected overexpression and knockdown S100A6 in 786-O, and caki-1 cell lines were developed by Cyagen Biosciences Company (China). The 2 vector maps and the efficiency of transfection were shown in Supplementary Figure 1. The stable transfected primers were shown in Supplementary Table S2.

### Cell proliferation assay

Cell growth analysis detected by MTS assay was described before [36].

### Subcutaneous xenograft mouse models

NOD/SCID mice aged 5 to 6 weeks were bred and maintained in compliance with NIH (National Institutes of Health) guidelines and were provided with veterinary care by full-time veterinary personnel at the Institutional Animal Care and Use Committee of the First Affiliated Hospital of the Chinese PLA General Hospital. For the *in vivo* tumor growth assay, the 786-O cells (5 × 10^6^) stably infected with shS100A, shControl, and untreated 786-O groups were suspended in PBS mixed with an equal volume of Matrigel (BD Biosciences USA) and 3 groups of mice, each consisting of 8, 8, and 6 mice, respectively were injected with the above mixture subcutaneously in the left armpit. After a period of 8 weeks, the mice were sacrificed after BrdU injection and tumors were excised. These tumors were suspended in 10% neutral formalin, and embedded in paraffin. The diameter and weight of tumors were measured. Hematoxylin-eosin (HE) staining was performed on 4 μm sections by routine procedures to identify the tumor lesions. Tumor size was measured using calipers, and volume was estimated by the following formula: volume = (length × width^2^)/2.

### RNA extraction and microarray analysis

The RNA extraction and the human genome arrays (Agilent Human (8*60K) were provided by the Agilent Technologies. The method of significance analysis of microarrays (feature extraction) was used to evaluate the significance of differences in gene expression. The ratio represented the gene expression alteration tendency between experimental and control groups. More than 2-fold changes in the gene expression were considered to be significant. To further define the biological process involving these different expression genes (DEGs), gene ontology (GO) enrichment analysis based on the DAVID database, the pathway analysis based on the KEGG database, and the genes interaction and network were searched in GeneMANIA [39, 40]. The result of microarray has been uploaded to Gene Expression Omnibus (Series GSE52708).

### RNAi knockdown

Three small interfering RNA (siRNA) duplexes targeting different coding regions of human CXCL14 and their scrambled sequence siRNA (mock) were customarily synthesized by Shanghai Gene-Pharma Co. (Shanghai, China). For the RNAi knockdown, equal numbers of cells were seeded in the plates containing medium without antibiotics for 24 h prior to the transfection. The siRNAs were introduced into the cells using Lipofectamine 2000 in serum-free Opti-MEM, according to the manufacturer's instructions. The expression levels of CXCL14 were determined after 72 h by western blot analyses (Figure 5C). The most efficient siRNA for knockdown was renamed as si-CXCL14 1# and 2#, the si-sequence were shown in Supplementary Table S2, chosen for further experiments, the scrambled sequence siRNA was named as si-scramble. The transfected cells were grown in complete medium at 37°C and 5% CO_2_. The cells were harvested at the indicated time points and used for further analysis.

### Cell cycle and apoptosis

Cell cycle and apoptosis analysis detected by flow cytometry were described before [36] and detailed in the Supplementary Data.

### Immunofluorescence

The cells were 4% formaldehyde fixed (10 min) and then incubated in 1%BSA/10% normal goat serum/0.3M glycine in 0.1% PBS-Tween for 1 h to permeabilise the cells and block non-specific protein-protein interactions. The cells were then incubated with the antibody ab46010 5 μg/ml overnight at +4°C. The secondary antibody (red) was Alexa Fluor® 488 goat anti-rabbit IgG (H+L) used at a 1/1000 dilution for 1 h. DAPI was used to stain the cell nuclei (blue) at a concentration of 1.43 μM.

### Statistical analysis

All values were shown as mean ± SD (standard deviation). For group comparison, paired *t* test (2 group comparison) or ANOVA (more than 2 group comparison) was done using the SPSS (version 12.0) software. Values with *P* < 0.05 were considered statistically significant.

## SUPPLEMENTARY MATERIALS AND METHODS


