# Supplementary material for: Elevated S100A6 (calcyclin) enhances tumorigenesis and suppresses CXCL14-induced apoptosis in clear cell renal cell carcinoma
Source: Oncotarget. 2015 Feb 3;6(9):6656–69. doi: 10.18632/oncotarget.3169 (PMC4466641; doi:10.18632/oncotarget.3169)
Supplement: Supplementary file 1 [file oncotarget-06-6656-s001.pdf]

SUPPLEMENTARY MATERIALS AND METHODS

Cell lines and reagents

The HKC and HK-2 cells were cultured in Dulbecco's modified Eagle's medium (HyClone). The 786-O and 769-P cells were cultured in RPMI 1640 medium (HyClone). The Caki-1 cells were cultured in MoCoy 5A medium (HyClone). The SN12-PM6 were cultured in DMEM with 1% MEM Vitamin Solution (Gibco) and 1% MEM Non-essential Amino Acid Solution (Gibco). Each culture medium was supplemented with 10% fatal bovin serum (FBS, Gibco). All cell lines were maintained at 37°C with 5% CO_2.

RNA isolation and real-time RT-PCR

Total RNA was extracted using the PARIS TM Kit (Applied Biosystems, Foster City, CA) according to the manufacturer's protocol. Multiscribe TM Reverse Transcriptase (Applied Biosystems) was used to synthesize the complementary DNA templates. Real-time reverse transcription–polymerase chain reactions were performed in an Applied Biosystems 7500 Detection system using MaximaH SYBR Green/ROX qPCR Master Mix Assays (Fermentas, USA). The expression of mRNA was determined from the threshold cycle (Ct), and the relative expression levels were normalized to the expression of human peptidylprolyl isomerase A (PPIA) mRNA (33) and calculated by the 2(-Delta Delta C(T)) method (34). Primers used in real-time RT-PCR were listed in Supplementary Table S1.

Protein extraction and western-blot analysis

Protein levels were quantified by Bradford assay. 30 mg protein from each sample was fractionated by 10% sodium dodecyl sulfate polyacrylamide gel electrophoresis and transferred onto polyvinylidene fluoride membranes (PVDF membranes, Millipore). The membrane was blocked in 0.1% Triton X-100 and 5% low fat milk powder in phosphate-buffered saline for 1 hour at 4°C and then probed with mouse monoclonal anti-glyceraldehyde-3-phosphate dehydrogenase (GAPDH) (1:1000; Abcam, ab9485), mouse monoclonal primary antibody, anti-Calcyclin(F-1) (1:500, Santa Cruz Biotechnology, sc-271396), rabbit polyclonal primary antibody, anti-CXCL14 (1:1000, Abcam, ab46010). After washing 3 times with Tris-buffered saline Tween-20, the membrane was incubated in peroxidase-conjugated goat anti-mouse/rabbit IgG antibody (1:1500, Santa Cruz Biotechnology). Bands were visualized by an enhanced chemiluminescence detection system using medical X-ray films and quantified by Photoshop (Adobe software). The intensities of band of interest were expressed relative to the GAPDH intensities from the same sample.

Immunohistochemistry

All samples were fixed in 10% neutral formalin. Sections were cut from wax blocks and mounted on APES-coated glass slides. Slides were dewaxed in xylene twice for 10 min and rehydrated in a graded ethanol series. Antigen retrieval was performed in 0.01 mol/L citrate buffer (Ph 6.0) by microwave oven for 2 min and 30 sec at 100°C. Endogenous peroxidase activity was blocked with 3% hydrogen peroxide for 15 min. After washing with phosphate buffered saline (PBS), sections with mouse monoclonal primary antibody, anti-Calcyclin(F-1) (1:200, Santa Cruz Biotechnology, sc-271396) in blocking solution was incubated at 4°C overnight in a humidified chamber. After washing three times with PBS, sections were incubated for 30 min with biotinylated secondary antibody (monoperoxidase-anti-mouse IgG, ZSGB-BIO). After washing in PBS 3 times, 3'-diaminobenzidine (DAB) was used as the chromogen. Slides were counterstained with hematoxylin for 5 min. Images were acquired with an OLYMPUS IX81 microscope (OLYMPUS, Japan). The images of tumor sections were taken at 100 × and 200 × magnification.

Cell proliferation assay

After treatment, the cells were plated in 96–well plates at a density of 2500 cells/well. On the 0, 24, 48, 72, 96 h of culture, the cells were washed and subjected to a CellTiter 96 ®AQueous One Solution Cell Proliferation Assay (Promega, USA), which was read at a wavelength of 490 nm using a 96–well ELx800TM Absorbance Microplate Reader (BiotekR, USA). Background 490 nm absorbance from the non–cellular control wells containing the same volumes of culture medium and CellTiter 96 ®AQueous One Solution Reagent was subtracted. All analyses were performed in triplicate on separate occasions. The mean and standard error at 490 nm absorbance of the experimental and control groups were analyzed at each of the aforementioned time points.

Cell cycle and apoptosis

The stable transfected cells were collected, and treated with 70% ethanol at 4°C overnight, and then washed with ice-cold PBS. For the detection of cell cycle, cell pellets were resuspended in RNase-containing (1:100 in dilution)PBS buffer in ice, and stained with propidium iodide (PI) (BD Biosciences, San Jose, CA) according to the manufacturer's protocol. Stained cells were analyzed on the FACS-Calibur (BD Biosciences). Data were analyzed using the Cellquest Pro software (BD Biosciences). Flow cytometry was performed to detect apoptosis with

AnnixinV-PE apoptosis Detection Kit (Beyotime). Cells were collected and resuspended in 0.5 ml PBS buffer. To these cells, Annixin V- PE were added lucifugal and incubated according protocol, and then analyzed by FCM (FACS- Calibur, Becton Dickinson, San Jose, CA).

RNAi knockdown

Three small interfering RNA (siRNA) duplexes targeting different coding regions of human CXCL14 and their scrambled sequence siRNA (mock) were custom synthesized by Shanghai Gene-Pharma Co. (Shanghai, China). For the RNAi knockdown, equal numbers of cells were seeded in the plates containing medium without antibiotics for 24 h prior to the transfection. The siRNAs were introduced into the cells using Lipofectamine 2000 in serum-free Opti-MEM, according to the manufacturer's instructions. The expression levels of CXCL14 were determined after 72 h by western blot analyses (Figure 5A). The most efficient siRNA for knockdown was renamed as si-CXCL14 1# and 2#, the si-sequence were shown in Supplementary Table S2, chosen for further experiments, the scrambled sequence siRNA was named as si-scramble. The transfected cells were grown in complete medium at 37°C and 5% CO_2.The cells were harvested at the indicated time points and used for further analysis.

eGFP Fluorescence

The eGFP fluorescence of the samples was detected and analyzed *in vitro* using a molecular imaging system (NightOWL II LB 983, Berthold, Germany). The signal intensity of the eGFP fluorescence from the tissues represented the amount of tumor lesions.

SUPPLEMENTARY FIGURES AND TABLES

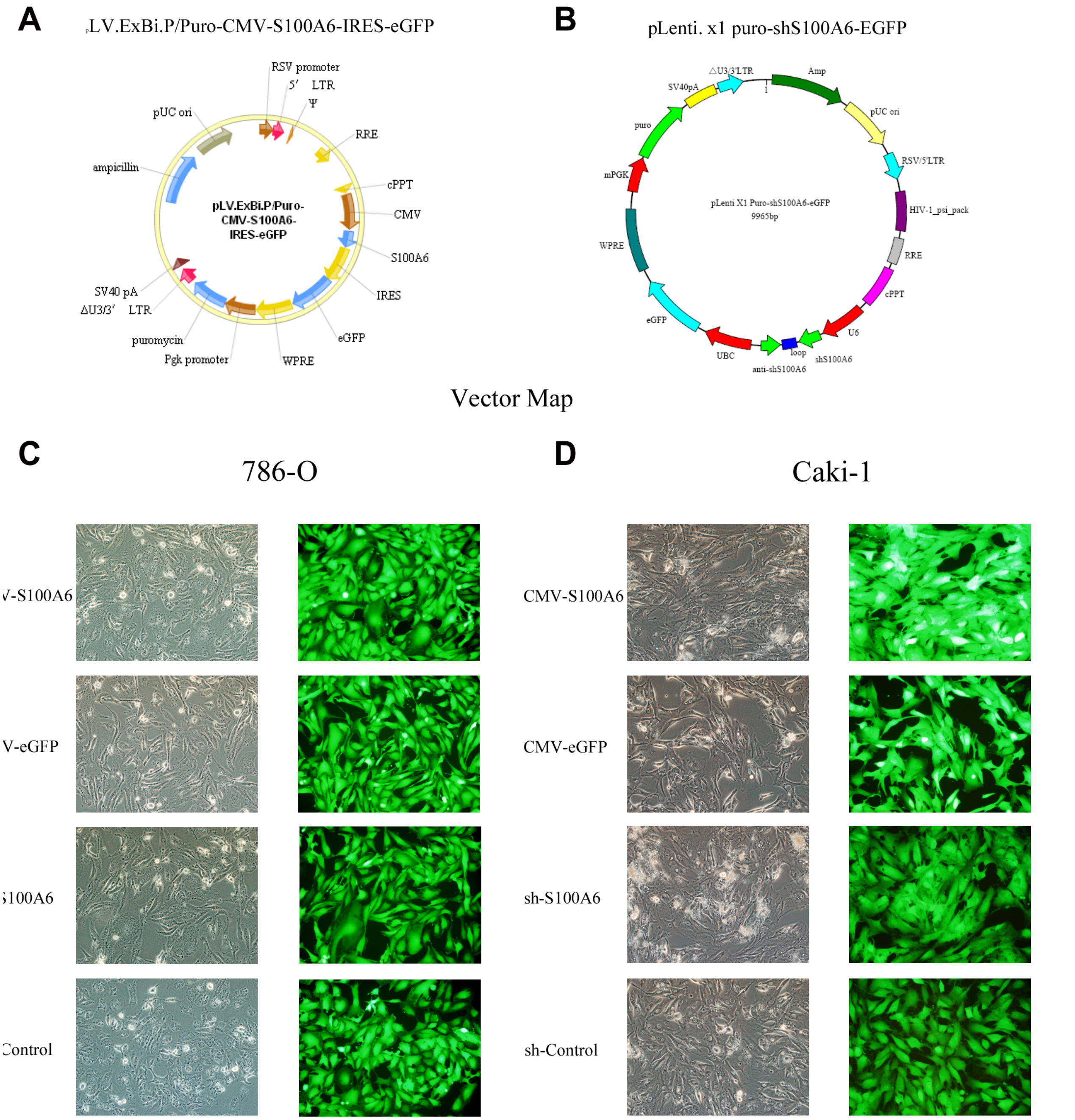

Supplementary Figure 1: (A) and **(B)** are the vector map of CMV-S100A6 and sh-S100A6. **(C)** is the transfection efficiency of overexpression and knockdown S100A6 in 786-O cell line. **(D)** is the transfection efficiency of overexpression and knockdown S100A6 in Caki-1 cell line.

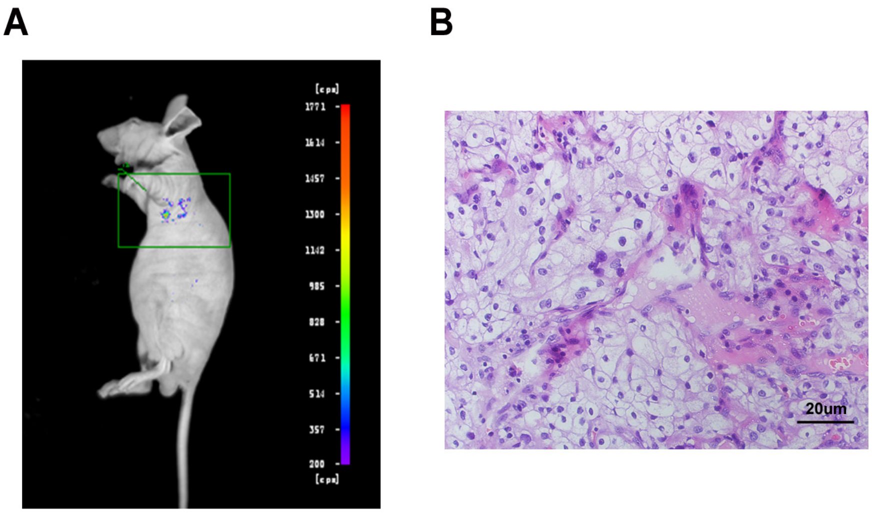

Supplementary Figure 2: (A) is the eGFP fluorescence of the subcutaneous xenograft mouse models. **(B)** is the HE stain of the tumor tissue.

Supplementary Table S1: All primers for real-time reverse transcription PCR assays

Primer name		Sequence	Product length (bp)
S100A6	Forward	CAAGAAGGAGCTGAAGGA(18)	143
	Reverse	CCAGGAAGGTGACATACT(18)	
CXCL14	Forward	GGAAATGAAGCCAAAGTAC(19)	182
	Reverse	TTTCACCCTATTCTTCGTA(19)	
PPIA	Forward	TCATCTGCACTGCCAAGACTG(21)	71
	Reverse	CATGCCTTCTTTCACTTTGCC(21)	

All primers for real-time reversion PCR were designed span the exon-exon junction.

Supplementary Table S2: sh/si RNA sequences against specific targets

Target Gene		Sequence
sh-S100A6	pLenti. x1 puro-shS100A6-EGFP	GCCTTGGCTTTGATCTACATTCAAGAGATGTAGATCAAAGCCAAGGC
1# si-CXCL14	Forward	GCGAGGAGAAGAUGGUUAUTT
	Reverse	AUAACCAUCUUCUCCUCGCTT
2# si-CXCL14	Forward	GCUUCAUCAAGUGGUACAATT
	Reverse	UUGUACCACUUGAUGAAGCTT
si-Scramble	Forward	UUCUCCGAACGUGUCACGUTT
	Reverse	ACGUGACACGUUCGGAGAATT

Supplementary Table S3: GO analysis of different expressed genes (DEGs) in the sh-S100A6& sh-Control group

Cluster	Terms and Transcriptional domain coverage (%)
**GO Biological Process**	Signal transduction (15.5%)
	Cell adhesion (9.3%)
	Proteolysis (7.9%)
	Ion transport (7.9%)
	Cell differentiation (7.3%)
	Blood coagulation (6.9%)
	Immune response (6.2%)
	Negative regulation of transcription from RNA polymerase II promoter (6%)
	Nervous system development (6%)
	Positive regulation of cell proliferation (5.8%)
	Cytokine-mediated signaling pathway (5.8%)
	Interspecies interaction between organisms (5.8%)
**GO Cellular Component**	Plasma membrane (51.5%)
	Extracellular region (39.9%)
	Extracellular spaces (19%)
	Proteinaceous extracellular matrix (7.2%)
	Microsome (6.2%)
	Cell surface (5.6%)
	Extracellular matrix (4.6%)
	Mitochondrial matrix (4.6%)
	Apical plasma membrane (4.3%)
	Axon (3.8%)
	Basement membrane (2.7%)
	Collagen (2.4%)
**GO Molecular Function**	Calcium binding (16.7%)
	Oxidoreductase activity (13%)
	Peptidase activity (11.8%)
	Protein homodimerization activity (11.8%)
	Receptor binding (6.8%)
	Transporter acivity (6.5%)
	Protein heterodimerization activity (6.2%)
	Growth factor activity (5.9%)
	Electron carrier activity (4.6%)
	Metalloendopeptidase activity (4.3%)
	Guanyl-nucleotide exchange factor activity (4.3%)
	Heparin binding (4%)

Cluster: A group of terms having similar biological meaning due to sharing similar gene members.

Supplementary Table S4: Pathway analysis of different expressed genes (DEGs) in the sh-S100A6&sh-Control group

Pathway	Transcriptional domain coverage (%)
Pathway in cancer	18.4%
Cytokine-cytokine receptor interaction	16%
Focal adhesion	12.3%
Chemokine signaling pathway	10.4%
Rheumatoid arthritis	9.2%
Neurotrophin signaling pathway	8.6%
Toll-like receptor signaling pathway	8.6%
Measles	8%
Insulin signaling pathway	8%
ECM-receptor interaction	8%
Metabolism of xenobiotics by P450	6.7%
Amoebiasis	6.7%
